# Acceleration of Osteogenesis via Stimulation of Angiogenesis by Combination with Scaffold and Connective Tissue Growth Factor

**DOI:** 10.3390/ma12132068

**Published:** 2019-06-27

**Authors:** Michiyo Honda, Ryo Hariya, Morio Matsumoto, Mamoru Aizawa

**Affiliations:** 1 Department of Applied Chemistry, School of Science and Technology, Meiji University, 1-1-1 Higashimita, Tama-ku, Kawasaki, Kanagawa 214-8571, Japan; 2 Department of Orthopedic Surgery, School of Medicine, Keio University, 35 Shinanomachi, Shinjuku-ku, Tokyo 160-8582, Japan

**Keywords:** osteogenesis, vascularization, CTGF, hydroxyapatite, scaffold

## Abstract

In bone regeneration, there are some important cellular biological processes, such as mineralization, cell organization, and differentiation. In particular, vascularization into regenerative tissues is a key step for the survival of cells and tissues. In this study, to fabricate biomimetic-engineered bone, including vascular networks, we focused on connective tissue growth factor (CTGF), a multifunctional protein which could regulate the extracellular matrix remodeling. By combination with CTGF and hydroxyapatite (HAp) ceramics (2D) or apatite-fiber scaffold (AFS, 3D), we have fabricated bioactive materials. The CTGF-loaded HAp ceramics could enhance the cellular attachment through interaction with integrin and promote actin cytoskeletal reorganization. CTGF-loaded HAp also enhanced the differentiation of osteoblasts by integrin-mediated activation of the signaling pathway. Under co-culture conditions, both osteoblasts and endothelial cells in the CTGF-loaded AFS were stimulated by CTGF, and each cell could penetrate the central region of the scaffold in vitro and in vivo. Direct cell-cell interaction would also improve the functionality of cells in bone formation. These results suggest that coupling between effective optimized scaffold and CTGF with multifunction could provide better mimicking natural bone by stimulation of angiogenesis.

## 1. Introduction

In bone tissue engineering, it is desirable that the scaffold could induce the osteogenesis and angiogenesis for enhancement of cell proliferation, differentiation, and matrix formation. In order to mimic native tissue environment, scaffolds with three-dimensional porous structure have been already fabricated by a variety of processes [1,2,3]. Calcium phosphates, a major component of bone, have been widely used for bone tissue engineering. In our previous studies, we have also demonstrated the biocompatibility and osteoconductivity of three-dimensional porous apatite-fiber scaffold [3,4].

Bone is a highly vascularized tissue and has a dynamic remodeling system with complex construction. The highly vascularized tissue is dependent on the spatiotemporal relationship between blood vessels and osteoblastic cells. Vascular networks are indispensable to supply cells with nutrients and oxygen for engineered tissue including bone after implantation. The lack of blood supply will be a major obstacle in tissue regeneration. Cells should induce angiogenesis around the tissue, via the newly formed vessel to deliver the nutrients, to establish a blood supply and remove the metabolic wastes. The importance of vascularization in the tissue engineering accelerates the development of regenerative tissue constructs including an organized vascular network [5,6]. For bone regeneration, various strategies, such as cell kinetics by growth factor delivery, co-culturing systems, application of mechanical stimulation, usage of biomaterials with suitable properties, and incorporation of microfabrication techniques, have been attempted [7]. Local delivery of growth factors affect the multiple cellular events, e.g., proliferation, migration, differentiation, and vascularization of an implanted graft [6]. A wide variety of growth factors are related to bone regeneration, such as bone morphogenetic protein-2 (BMP-2), transforming growth factor-β (TGF-β), fibroblast growth factor (FGF), platelet-derived growth factor (PDGF), insulin-like growth factor (IGF), endothelin-1, and vascular endothelial growth factor (VEGF) [6]. As shown in previous studies, scaffolds loaded with BMP-2 and VEGF accelerated not only bone formation also vascularization [8,9]. In another study, a synergistic effect was produced by combination with TGF- β and BMP-7 in new bone formation [10]. Both angiogenesis and osteogenesis are an essential component of bone regeneration to mimic the native environment.

Connective tissue growth factor (CTGF) is known as the CCN family of matricellular proteins. CTGF is a cysteine-rich protein of secreted via a 37-amino acid signal sequence. CTGF has been shown to regulate multicellular functions. In addition, CTGF can be involved with various biological processes, such as angiogenesis, chondrogenesis, and osteogenesis [11,12,13]. In osteoblasts, expression levels of CTGF were upregulated by BMP, TGF-β and Wnt. Furthermore, CTGF regulates different cellular functions, including cellular attachment, growth, migration and differentiation [11,14]. That is, CTGF is an excellent growth factor that can simultaneously satisfy osteogenesis and angiogenesis.

To develop engineered constructs with the enhancement of the vascular networks and bone formation, we focused on CTGF and fabricated CTGF-loaded 2D or 3D bioactive materials. We then explored the effects of CTGF on osteogenesis and evaluated in vitro and in vivo responses. Combination with scaffold and CTGF could make it possible to enhance angiogenesis and stimulate bone formation including cellular functions.

## 2. Materials and Methods

### 2.1. Material Preparation

The hydroxyapatite (HAp) powders (HAp-100, Taihei Chemical, Japan) were used for fabrication of HAp ceramics. To obtain HAp ceramics, HAp powders (HAp-100) were uniaxially compressed at 50 Mpa in disks (diameter: ~20 mm, thickness: ~2.0 mm) and sintered at 1200 °C for 5 h at a heating rate of 10 °C·min^−1^. To fabricate CTGF-HAp ceramics, the HAp ceramics were coated with recombinant CTGF (0, 50, and 100 ng; R&D Systems, Minneapolis, MN, USA) in sterile water using 0.1% bovine serum albumin (BSA) solution as a binding agent. Hereafter, HAp ceramics coated with CTFG are referred to as “CTGF-HAp(x)” depending on CTGF amount.

Apatite-fiber scaffolds (AFSs) were fabricated according to the previous reports [15,16]. In brief, the apatite fibers (Afs) were prepared by a homogeneous precipitation method using urea in aqueous solutions of Ca(NO_3_)_2_-(NH_4_)_2_HPO_4_-(NH_2_)_2_COHNO_3_. The fibers with long-axis sizes of about 100 µm were composed of carbonate-containing apatite with preferred orientation along the c-axis. Afs were mixed with spherical carbon beads (Nika beads; Nihon Carbon Company, Tokyo, Japan) with a diameter of both 20 and 150 µm (at a ratio of 1:1) in the solvent (ethanol/water = 1/1 (*v*/*v*)) at a 3/1 of 5/1 carbon/ Afs (*w*/*w*) ratio. The green compacts were prepared by uniaxially-pressing at 30 MPa in metal molds. The resulting compact was fired at 1300 °C for 5 h in a water-vapor atmosphere. The two AFSs with different dimensions were used for in vitro (diameter: ~15 mm × thickness: ~2.0 mm) and in vivo (~4.0 mm × ~8.0 mm) evaluations. To prepare the CTGF-AFS, scaffolds were loaded with CTGF solutions (50, 100 ng for in vitro and 1, 5 μg for in vivo) by vacuum infiltration technique. On the other hand, the AFS dropped 0.1% bovine serum albumin (BSA; Sigma-Aldrich, St. Louis, MO, USA) -H_2_O solution was used as a control. The scaffolds are denoted according to the concentration of CTGF and ratio of Afs/CBs; for example, 50 ng CTGF-loaded AFS (AF and CB at a ratio of 1:3 (*w*/*w*)) is denoted as “CTGF(50)-AFS300”. The identity of crystalline phases of the Afs and AFSs were determined by X-ray diffractometry (XRD; MiniFlex, Rigaku Co., Tokyo, Japan) with Cu-K radiation generated at 30 kV and 15 mA. Data were collected in the 2θ range of 4–50° with a step size of 0.04° and at a speed of 4°/min. The JCPDS reference patterns use the identification of crystalline phases for HAp(#9-0432). The morphology of the scaffolds was observed by scanning electron microscopy (SEM; JSM-6390LA, JEOL, Tokyo, Japan) at an accelerating voltage of 15 kV. Samples for SEM observation were prepared by coating with Pt using sputtering before SEM observation.

### 2.2. Cell Culture

Osteoblast-like MC3T3-E1, MG-63 cells (European Collection of Cell Culture) and human umbilical vein endothelial cells (HUVECs, PromoCell, Heidelberg, Germany) were cultured in the present study. MC3T3-E1 cells were cultured in alpha-modified minimal essential medium (MEMα; Sigma-Aldrich, St. Louis, MO, USA) with 10% of fetal bovine serum (FBS; Sigma-Aldrich, St. Louis, MO, USA). MG-63 cells were cultured in Eagle’s minimal essential medium (EMEM, Sigma-Aldrich, St. Louis, MO, USA) supplemented with 2 mM glutamine (Sigma-Aldrich, St. Louis, MO, USA), 1% non-essential amino acids (Sigma-Aldrich), and 10% of FBS. HUVECs were cultured in Endothelial cell growth medium 2 (EGM2; Takara Bio Inc., Shiga, Japan). All cells were maintained at 37 °C in a humidified atmosphere containing 5% CO_2_. Under co-culture conditions, both MG-63 cells and HUVECs were seeded in each AFS set on a 24 well culture plate in EGM2 at an initial cell number of 2 × 10^5^ cells and 8 × 10^5^ cells, respectively. As a control, we seeded MG-63 cells at the same cell numbers and cultured in EMEM medium.

### 2.3. Cell Adhesion Assay

The MC3T3-E1 cells were seeded on the HAp disks set on a 24 well culture plate at a density of 1.0 × 10^5^ cells/well in 1 mL of MEMα. Cells were maintained at 37 °C, 5% CO_2_ in a humidified atmosphere. At 5 and 24 h after incubation, each disk was rinsed with phosphate-buffered saline (PBS) twice. Efficacy of cell attachment is calculated by the ratio between the number of adhering cells to the disks at 5 h and the total number of adhered cells on the control of the tissue culture plate. Therefore, the value shows the initial attachment of cells/substrate. Quantification of viable cells on the specimens was carried out using hemocytometer. Experiments were repeated three times and run in triplicate.

### 2.4. Cell Proliferation

The MC3T3-E1 cells were seeded onto the disks at a density of 1.0 × 10^5^ cells/well in a 24 well culture plate in 1 mL of MEM α. The culture was maintained at 37 °C, 5% CO_2_ in a humid atmosphere. The culture medium was changed every two days, and the culture was continued for a maximum of seven days. The number of proliferating cells was counted at day 1, 3, 5, and 7 after plating. Experiments were repeated three times and run in triplicate.

### 2.5. Immunostaining

Cells were grown on the ceramics or AFSs washed with PBS (pH 7.4), fixed with 4% paraformaldehyde/PBS for 15 min. After that, cells were permeabilized with 0.1% Triton X-100/PBS (pH 7.4) for 15 min. Cells were rinsed with PBS, and then stained with Alexa Fluor^®^488-labeled phalloidin for F-actin at 20 °C for 1 h or with vinculin (Sigma-Aldrich, St. Louis, MO, USA) or CD31 (Dako, Santa Clara, CA) monoclonal antibody at 4 °C overnight. Alexa Fluor^®^594-conjugated secondary antibody was used (Thermo Fisher SCIENTIFIC, Waltham, MA). Nuclei were stained with 4′, 6-diamidino-2-phenylindole (DAPI, Dojindo, Kumamoto, Japan). Cells were again washed with PBS, and then examined with fluorescence microscopy (FV300 and BX51, Olympus, Tokyo, Japan). Cell size (spreading area) was measured using ImageJ software [Version 1.52o, National Institutes of Health (NIH), Bethesda, MD, USA]. Experiments were repeated three times and run in triplicate.

### 2.6. Bone Differentiation

Histochemical staining for alkaline phosphatase (ALP) activity was performed using a commercially available kit (FUJIFILM Wako Pure Chemical, Osaka, Japan) according to the manufacturer’s instructions. ALP activity was also performed on days 7 and 14. On these days, the cellular proteins were solubilized with CelLytic^TM^ M (Sigma-Aldrich) and centrifuged. The supernatants were assayed for ALP activity (FUJIFILM Wako Pure Chemical, Osaka, Japan) according to the manufacturer’s instructions. Enzymatic activity was normalized to total protein concentration using BSA. The measurement of protein was done by using the standard method of Bradford (FUJIFILM Wako Pure Chemical, Osaka, Japan). Experiments were repeated three times and run in triplicate. Cells were evaluated for calcium production at 28 days by staining with 10% Alizarin Red solution (Sigma-Aldrich, St. Louis, MO, USA). Alizarin Red S is a dye that binds to calcium salts. After fixation and staining cells were again washed and examined with light microscopy. Quantification of staining intensity was measured using the ImageJ software.

### 2.7. In Vivo Study

#### 2.7.1. Angiogenesis in Rats

In vivo study was conducted on four male Wister rats (age: Four-week-old, body weight: 200–220 g). The animals were anesthetized with isoflurane. A dorsal shaving was performed, and the area was disinfected with an iodine solution. A 2cm incision was made in the backs of the rats, in a head-to-tail alignment orientation. The four specimens were implanted into the subcutaneous tissue of the rat. After two weeks of the implantation, the samples and surrounding tissues were retrieved from the rat. The living reaction was examined by histological evaluation, such as hematoxylin and eosin (H&E), immunostaining of vascular endothelial growth factor (VEGF) and alpha-smooth muscle actin (SMA). Antigen was detected using primary antibody, such as monoclonal rat anti-CD31 (Bio-Rad, Hercules, CA, USA) and polyclonal rabbit anti-VEGFA (Abcam, Cambridge, UK) in conjunction with an HRP/DAB detection kit (Abcam, Cambridge, UK) according to the manufacturer’s instructions. Animal experiments were approved by the Animal Care and Use Committee of Meiji University (AEFST2017-007).

#### 2.7.2. Bone Formation in Rabbits

In vivo study was conducted on three Japan White rabbits weighing about 3.0 kg (16 weeks, male) for evaluation of the biocompatibility and osteoconductivity of CTGF-AFSs. Surgery was performed under general anesthesia. The tibia of a rabbit was exposed, and cylindrical defects (4.4 mm in diameter) were drilled in the epiphysis of the tibia. Specimens were inserted into the defect for eight weeks. The study was performed in six tibias (of three rabbits) into which cylindrical implants were inserted. After implantation, rabbits were sacrificed using sodium pentobarbital and specimens together with surrounding tissue were removed. Decalcified specimens were fixed in 4% paraformaldehyde/PBS solution (pH 7.4). After that, samples were decalcified in a mixture of formic acid and sodium citrate. The samples were embedded in paraffin, cut into 5–8 µm serial sections, and stained with H&E. The sections were histologically observed by microscopy (IX71, Olympus, Tokyo, Japan). Quantification of bone formation area was measured using the NDP view software. Five points from each specimen were chosen at random. Undecalcified histological specimens were fixed in 70% ethanol. After that, samples were dehydrated in an alcohol solution, defatted and embedded in methyl-methacrylate resin. Finally, samples were cut sections by a microtome. The specimens were stained with Villanueva bone stain. All animal experiments were followed by the guideline of the Animal Care and Use Committee of Keio University (09067-(11)).

### 2.8. Statistical Analysis

The data were statistically analyzed for determination of the mean and the standard deviation of the mean. The Student’s *t*-test was carried out with a significance level of *p* < 0.05.

## 3. Results

### 3.1. CTGF Enhanced Cellular Attachment and Cell Spreading

**Figure 1:**
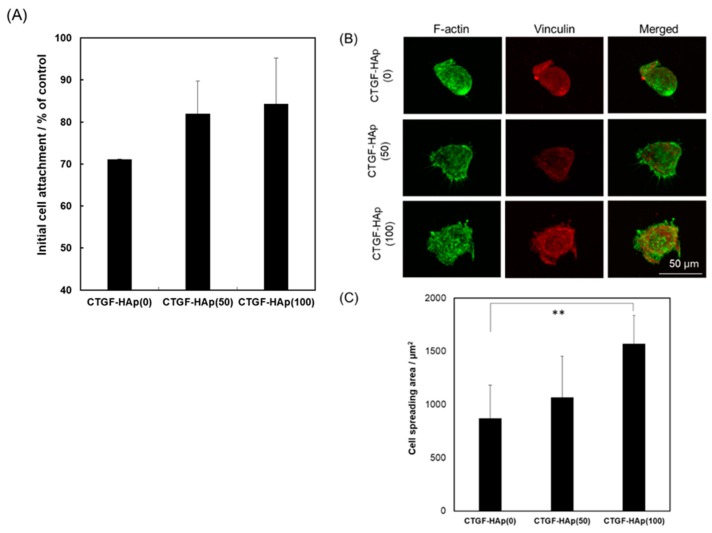
Effect of connective tissue growth factor (CTGF) on cellular attachment and morphology at 5 h after seeding. (**A**) Initial cell attachment as a percentage of the tissue culture plate. CTGF-HAp(100) ceramic has the highest percentage of the attached cell. Cell attachment increased with CTGF, though there were no significant differences among them. (**B**) Fluorescence images of MC3T3-E1 cells on CTGF-HAp ceramics at 5 h. Actin filaments were stained with Alexa Fluor^®^488-labeled phalloidin (green), and vinculin was stained with monoclonal anti-vinculin (red). Scale bar indicates 50 μm. (**C**) Cell spreading was quantified by measuring the area of cells from fluorescent images of cells. Bar graphs represent means ± SD (n = 13). ** Significantly different (*p* < 0.001) from the value with CTGF-HAp(0).

The initial cell attachment is an important step for subsequent behavior of cells. The interaction between materials and cells contributes to the success of regenerative therapeutics. Cells were seeded on CTGF-loaded HAp ceramics to examine the effect of CTGF on cellular attachment to the substrate. At 5 h after seeding, cells were detached from each ceramic and counted by hemocytometer. As a result, the higher cellular attachment can be attributed to the increase with CTGF concentration (Figure 1A). However, there were no significant differences among each disk. After 24 h immersion in the culture medium, we could detect a small amount of CTGF, which was not enough to promote cellular attachment (Figure S1) [17]. Furthermore, to assess the ability to promote cell adhesion through the integrin by CTGF, we explored actin cytoskeleton. We could see cells with a large spreading area per individual cell on the CTGF-loaded HAp ceramics, especially CTGF-HAp(100), while cells on HAp ceramics had a small spreading area and a more rounded morphology (Figure 1B,C). These results suggest that immobilized CTGF on HAp ceramics could enhance cellular attachment through interaction with integrin [18].

**Figure 2:**
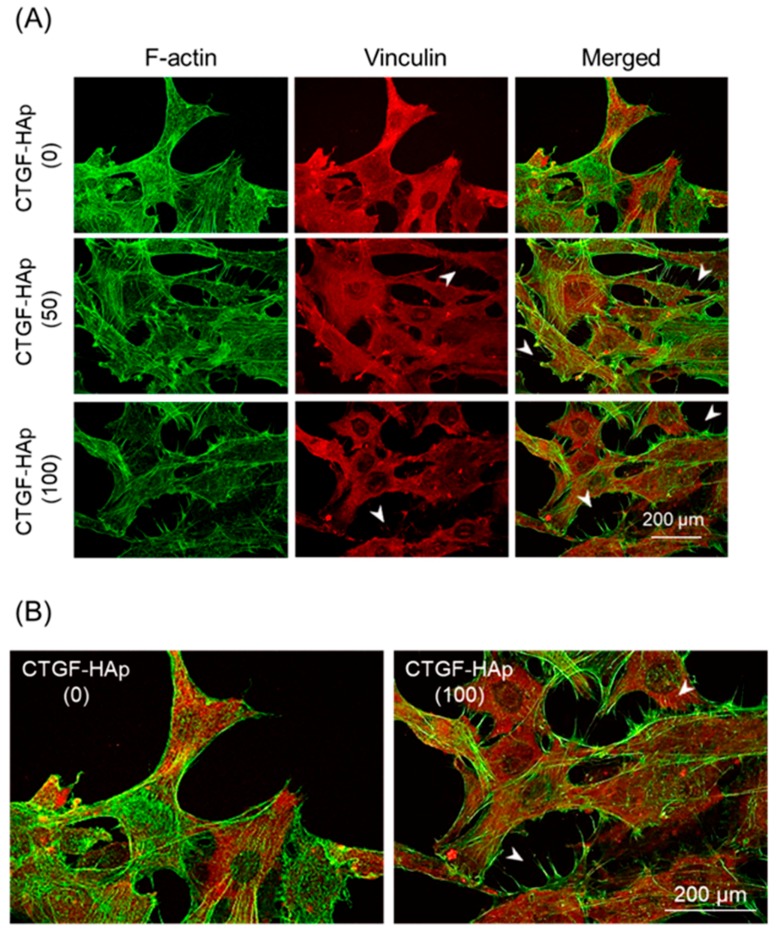
Immunostaining and fluorescence images of cell attachment at 24 h after plating. (**A**) Actin filaments were visualized with Alexa 488 phalloidin (green). Cells on CTGF-HAp ceramics were also immunostained with primary antibody against vinculin, followed by Alexa Fluor^®^594-conjugated secondary antibody. (**B**) Comparison with CTGF-HAp(0) and (100), regions in Figure 2A (merged) are magnified as insets. Arrowheads indicate filopodia-like structure. Bars represent 200 μm.

**Figure 3:**
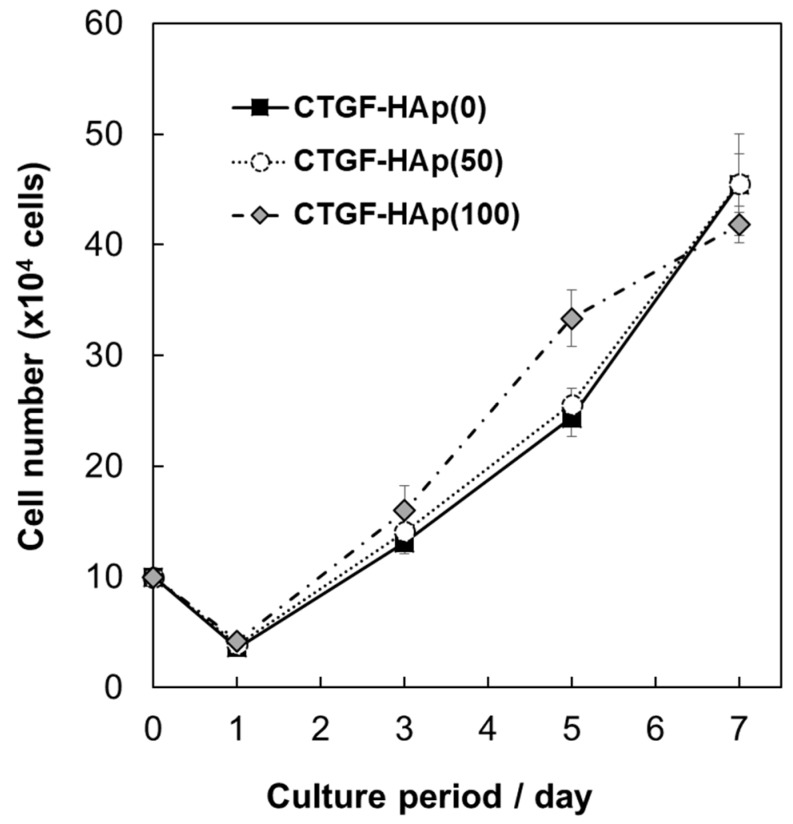
Cell proliferation on CTGF-loaded HAp ceramics. To examine the effect of proliferation on osteoblasts by CTGF, cells were cultured on CTGF-HAp ceramics for seven days. The number of proliferating cells was counted at day 1, 3, 5, and 7 after plating. Experiments were repeated three times and run in triplicate. Graphs represent means ± SD.

To confirm whether loading CTGF will facilitate cellular attachment and spreading, we imaged cells at 24 h of culture on HAp ceramics with/without CTGF (Figure 2). At 24 h after seeding, cells were well-spread on all ceramics. Above all, filopodia, thin, finger-like and highly dynamic actin-rich membrane protrusions, could be observed on only CTGF-HAp ceramics (Figure 2B). These results indicate that CTGF could promote actin cytoskeletal reorganization, such as cell spreading and membrane protrusion. Next, to examine the effect of proliferation on osteoblasts by CTGF, cells were cultured on CTGF-HAp ceramics for seven days. As a result, no obvious changes were seen among these samples (Figure 3). These data suggest that CTGF did not influence cell proliferation. These results agreed with previous studies [17,19].

### 3.2. CTGF Enhanced Osteogenic Differentiation

**Figure 4:**
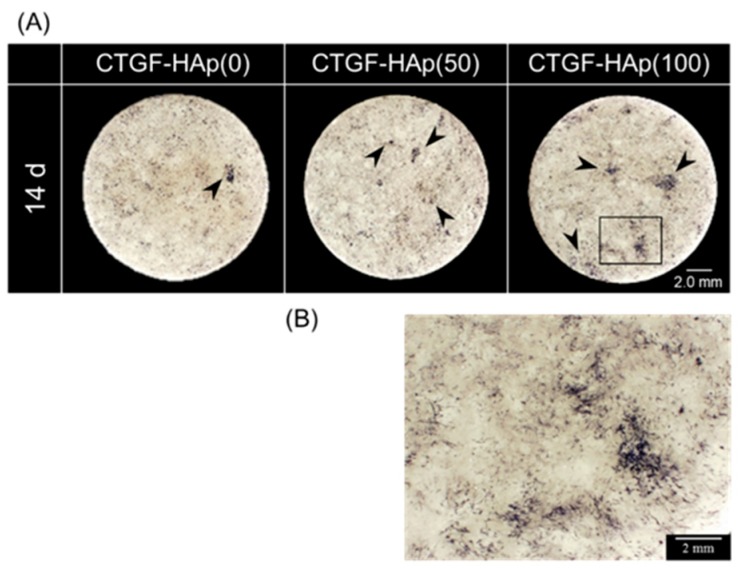

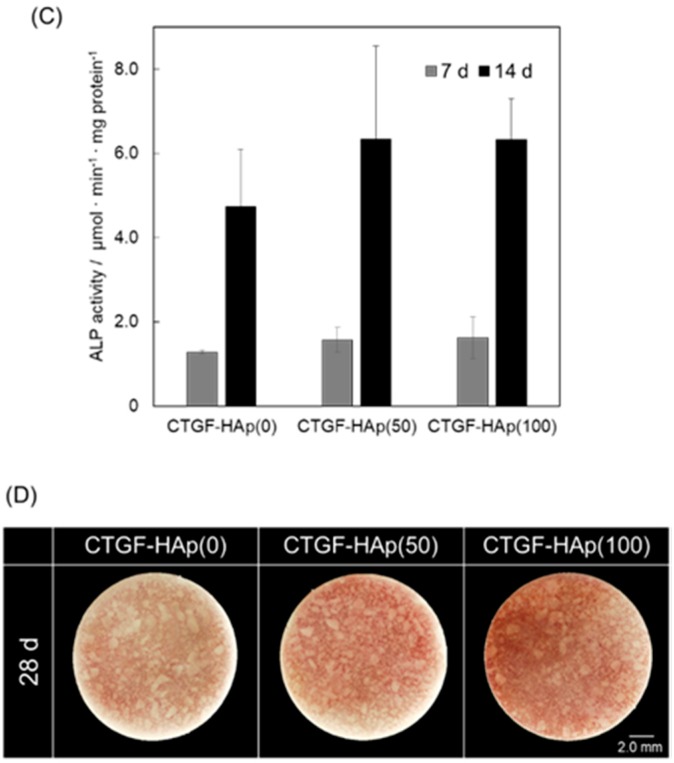
Comparison of osteogenic differentiation between CTGF-loaded ceramics and HAp ceramics (control). (**A**) Cells on CTGF-HAp ceramics were stained for ALP levels after culturing for 14 days. Arrowheads represent cell cluster with high ALP activity. (**B**) CTGF increased ALP activity in clustered regions (Higher magnification (square in A) of MC3T3-E1 on CTGF-HAp(100) ceramics). (**C**) Comparison of ALP activity of cells at day 7 and 14 in culture. Loading CTGF increased ALP activity. Experiments were repeated three times and run in triplicate. Graphs represent means ± SD. (**D**) Cells on CTGF-HAp ceramics were cultured for 28 days, and calcium deposition was assessed by Alizarin red staining.

Previous studies showed that CTGF enhanced osteogenesis and chondrogenesis [14]. To investigate the efficacy of osteogenic ability by loading CTGF, MC3T3-E1 cells were cultured on CTGF-HAp ceramics for 14 or 28 days and stained with alkaline phosphatase (ALP) or Alizarin Red. We observed some cell clusters with strong staining on the CTGF-HAp(50, 100) ceramics; whereas, there were cells with very weak staining in the control culture (CTGF-HAp(0)) (Figure 4A). Especially, CTGF-HAp(100) increased ALP activity in cluster regions (Figure 4B). Additionally, ALP activity was also measured on day 7 and 14 (Figure 4C). The ALP activity of cells cultured on each ceramic significantly increased at day 14. In particular, cells cultured on CTGF-HAp(50, 100) had a higher ALP activity than control. However, statistical differences were not seen among the three samples. Furthermore, to evaluate calcium deposits in cell culture, cells were then stained with alizarin red solution.

Alizarin red staining also showed that more mineralized nodules on CTGF-HAp(100) ceramics than on CTGF-HAp(0) ceramics (Figure 4D). All ceramics were analyzed by using ImageJ software to quantify the area of mineralization to quantify the mineralized area. Quantification of staining intensity showed that mineralized area of CTGF-HAp (50 and 100) ceramics were 6.6 and 8.3 times higher than that of control, respectively. These data demonstrated that two-dimensional CTGF-HAp ceramics could enhance osteogenic differentiation.

### 3.3. CTGF Enhanced Cell Penetration into 3D Scaffold

**Figure 5:**
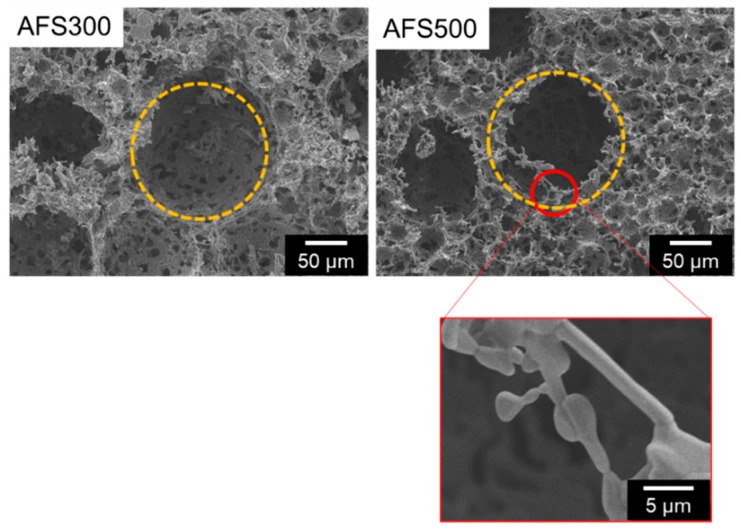
SEM images of AFS300 and AFS500 with two types of pores. Yellow circles indicate macro pores and red one represents micro pores. Scale bars represent 5, 50 μm, respectively.

**Figure 6:**
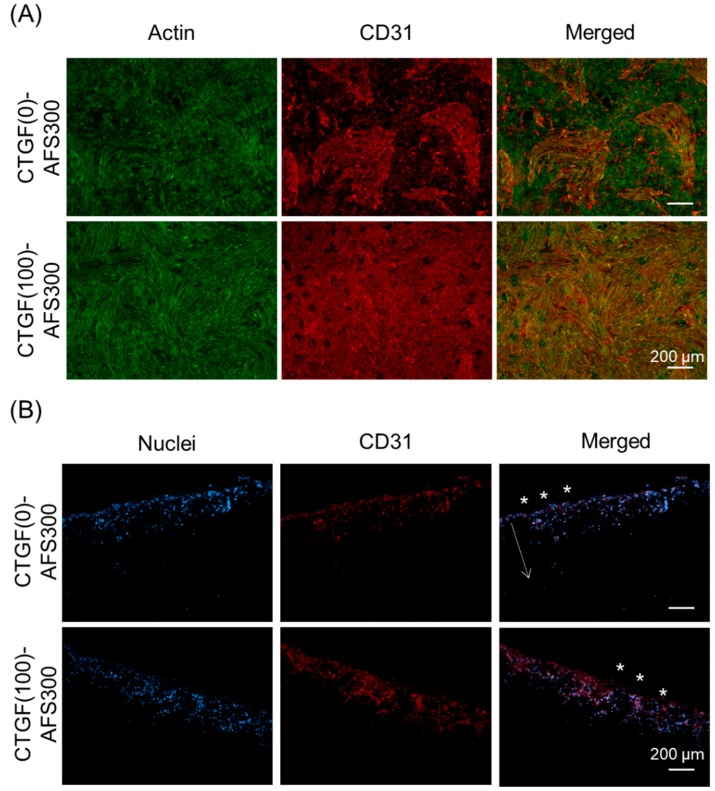
Immunostaining and fluorescence images of cellular distribution and penetration in AFS300. (**A**) Two kinds of cells (MG-63 and HUVEC) were co-cultured in three-dimensional porous scaffold for 14 days. F-actin was visualized with Alexa Fluor^®^488-labeled phalloidin (green). CD31 as a marker of endothelial cells was immunostained with primary antibody against CD31, followed by Alexa Fluor^®^594-conjugated secondary antibody (red). Experiments were repeated three times and run in triplicate. (**B**) MG-63 and HUVEC were co-cultured in AFS300 for 14 days. Samples were immunostained with CD31 (red fluorescence, endothelial-specific) and nuclei were counterstained with DAPI (blue fluorescence, MG-63 and HUVECs). The Z-axis images show the three-dimensional distribution of cells. Asterisks represent the surface of scaffold and cells penetrated the inner part of the scaffold, as shown by the arrow. Scale bars indicate 200 μm. Experiments were repeated three times and run in triplicate. The distances from the surface of CTGF(0)-AFS300 or CTGF(100)-AFS300 were measured by ImageJ (Figure S3).

A three-dimensional apatite fiber-scaffold (AFS) was fabricated to construct engineered bone with a biomimetic environment. The properties of the AFSs represent in Table 1. The porosity increased depending on the additional number of CBs. On the other hand, the compressive strength decreased depending on the porosity. XRD patterns show that all samples were HAp single phase despite the additional number of CBs (data not shown). Figure 5 illustrates the microstructural observation by SEM. We could observe two kinds of pores: Macro pores due to CBs burned out, and micro pores due to the intertwining of the individual fibers. Macro pores may affect the cell invasion, and micro pores also may contribute to the supply of body fluid. To regenerate engineering bone tissues, with their complicated cell structures, we designed biomimetic scaffold by combination with co-culture system and growth factor-loaded scaffold [20]. In this study, two kinds of cells (MG-63 and HUVEC) were co-cultured in three-dimensional porous scaffold incorporated with CTGF. At first, we have examined the effects of CTGF on cell distribution and penetration by observation of their morphology. Cells in AFSs were co-cultured for 14 days and were discriminated between osteoblasts and endothelial cells by immunostaining with CD31 as a marker of endothelial cells. The images show that cells in both AFSs were fully extended and formed bridges within macro pores at the surface of scaffolds (Left panels in Figure 6A). However, the number of HUVECs in CTGF(100)-AFS300 was much higher than that in CTGF(0)-AFS300 (Center panels in Figure 6A). These results suggest that CTGF promoted cell migration and, subsequently, direct cell to cell contact. Additionally, to assess the ability of an organized vascular network formation, cell penetration into scaffolds was explored (Figure 6B). Fluorescence images showed that CTGF could induce migration of both osteoblasts and vascular endothelial cells. Image analysis revealed that cells in CTGF(100)-AFS300 reached a depth of 200–400 μm from the surface of the scaffold. On the other hand, cells in CTGF(0)-AFS300 penetrated at a depth of 40–200 μm from the surface. These data suggest that CTGF could enhance cell migration (Figure S3). Similar trends were observed in AFS 500. However, cell invasiveness increased depending on porosity (Figure S4). Focused on HUVECs, HUVECs were co-localized with osteoblasts. These results suggest that cell-cell interaction with endothelial cells and osteoblasts could promote cell proliferation and induction of vascular networks. The release kinetics of CTGF from AFS into PBS up to 14 days were also examined (Figure S2). The initial burst of CTGF was detected in the first 24 h. However, the release rate gradually decreased. The cumulatively released CTGF was equivalent to approximately 5% of the total amount for each CTGF-AFS after 14 days of incubation. The difference between ceramics and scaffold might contribute to the adsorption of CTGF to HAp.

### 3.4. CTGF Enhanced In Vivo Angiogenesis and Bone Formation

**Figure 7:**
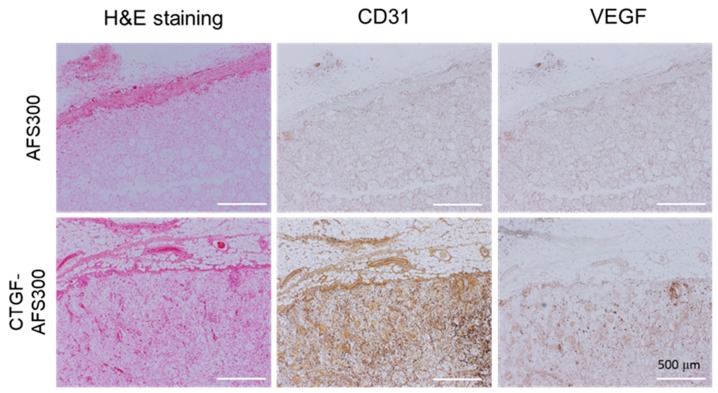
Immunohistochemical analysis of angiogenesis. H&E staining was performed on tissue sections. Thin sections were also immunostained with CD31 and VEGF as a marker of endothelial cells (Brown DAB staining). Cells were penetrated both AFS and CTGF-loaded AFS. CTGF(1000)-AFS300 displayed a higher density of cells including vessels. Scale bars indicate 500 μm, respectively.

Under in vitro co-culture condition, CTGF could stimulate both osteogenesis and angiogenesis. At first, we evaluated the ability of angiogenesis by CTGF-loaded AFSs in vivo. Samples were implanted into the subcutaneous tissue of rats for two weeks and employed for histological evaluation. As a result, H&E staining showed that cells penetrated macro pores, and well-proliferated in the CTGF(1000)-AFS300 (Figure 7, H&E). Depending on the porosity of the scaffold, many cells that invaded AFS were observed (Figure S5). On the other hand, to assess the ability of angiogenesis by CTGF-loaded AFS, angiogenesis specific markers (CD31 and VEGF) were examined by immunostaining. Immunohistochemistry clearly showed that the expression of CD31 in CTGF(1000)-AFS300 increased dramatically compared to AFS300. We could also observe numerous VEGF-positive cells in CTGF-loaded AFSs. These results suggest that the vascularization in CTGF-loaded AFSs was significantly enhanced, compared to the control group of scaffolds.

**Figure 8:**
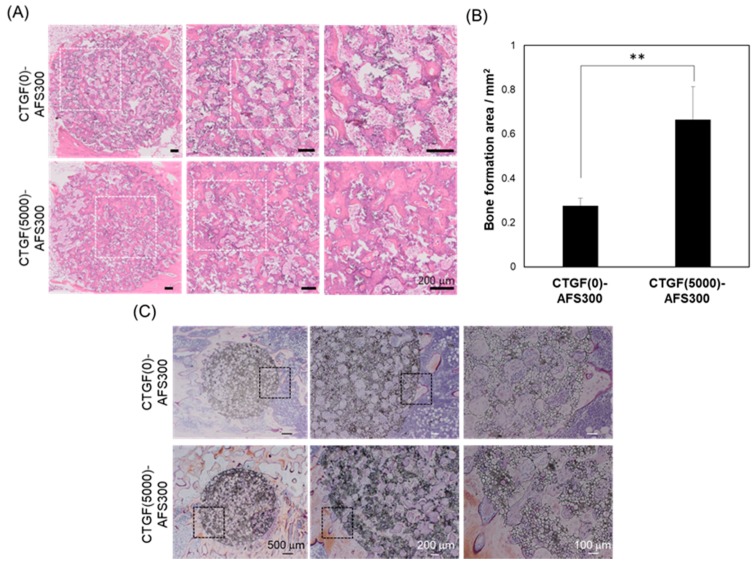
Photomicrographs of histological sections stained with H&E and Villanueva bone staining in CTGF(0)-AFS300 and CTGF(5000)-AFS300, eight weeks after the operation. (**A**) In contrast to the CTGF(0)-AFS300, CTGF(5000)-AFS300 contained numerous cells. New bone formation was observed in CTGF-loaded AFS. Scale bars indicate 200 μm, respectively. (**B**) The newly-formed bone of CTGF(0)-AFS300 or CTGF(5000)-AFS300 were measured by NDP view software. Bar graphs represent means ± SD. ** Significantly different (*p* < 0.01) from the value with CTGF-HAp(0). (**C**) Upper panels showed CTGF(0)-AFS300 and lower panels represent CTGF(5000)-AFS300. Center panels show high magnification of the square area in left panels. Right panels represent high magnification of the square area in center panels. Scale bars indicate 500, 200, 100 μm, respectively.

**Figure 9:**
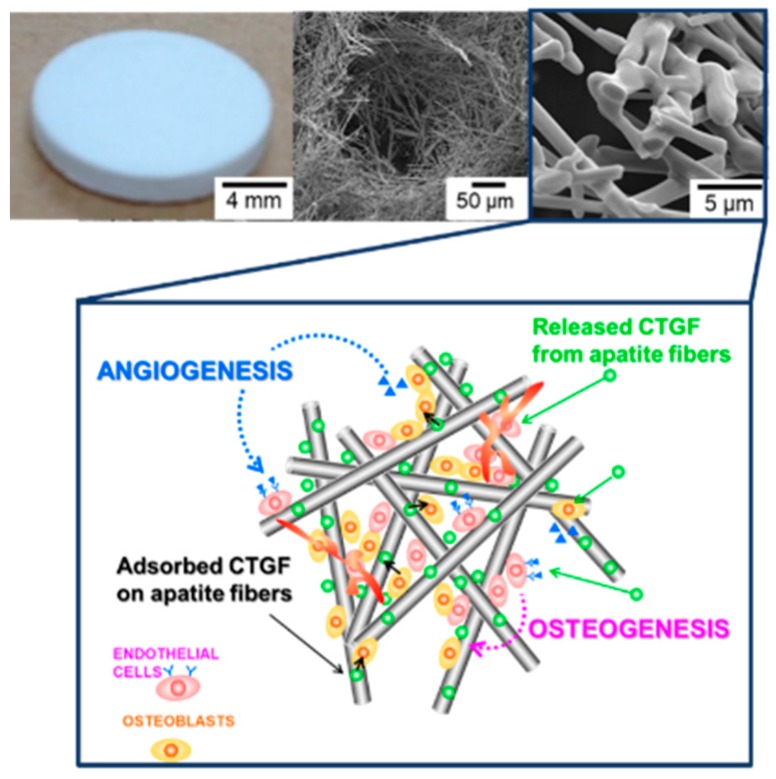
Schematic diagram of CTGF-loaded AFS. Schematic diagram of the relation between osteogenesis and angiogenesis by CTGF stimulation. Both released and adsorbed CTGF might stimulate osteoblasts and endothelial cells.

Next, to examine the ability of bone formation by CTGF-loaded AFSs in vivo, these samples were then implanted into rabbit tibiae for eight weeks. As shown in Table 1, the porosity of AFS500 was over 90%, and the compressive strength was under 0.01 MPa. Therefore, we selected AFS300 for in vivo test. After implantation, the specimens were stained with H&E staining or Villanueva bone stain. Histological analysis by H&E staining showed that the newly-formed bone fulfilled with defects in CTGF(5000)-AFS300 compared to CTGF(0)-AFS300 (Figure 8A). In both scaffolds, cells penetrated macro pores and pores were filled with cells. However, the density of the extracellular matrix (ECM) had increased, and the area of newly-formed bone in CTGF(5000)-AFS(300) was about twice as large as that in AFS only (Figure 8B). These data demonstrated that CTGF plays an important role in enhancing bone regeneration. Since the amount of bone formation depends on the implant site, further in vivo evaluation is required. Furthermore, to evaluate the bone formation of CTGF-loading AFS, Villanueva bone staining was performed (Figure 8C). The levels of bone ingrowth through scaffolds with or without CTGF was examined. Tibiae stained using Villanueva bone staining method showed bone tissues-to-AFSs direct bonding without causing damage to surrounding tissues. Villanueva bone staining has also been shown that the area of calcified bone (as shown by brown color) around CTGF-AFS was much larger than that around AFS only. These results indicate that CTGF-AFS promotes bone ingrowth and plays a critical role in not only cell proliferation and differentiation, but also vascularization. CTGF released from AFS would regulate cell proliferation, migration and cell-cell interaction during osteogenesis (Figure 9). In addition, these data revealed that porous AFSs could provide sufficient space for osteoblastic cell proliferation and differentiation and incorporate with growth factors for successful development of bone tissue.

## 4. Discussion

At the central region of the replacements, larger engineered tissues lead to necrosis due to the lack of blood supply. To overcome these problems, a variety of strategies have been addressed to construct the vascular networks within implanted tissues for the achievement of successful integration with the host tissue [21,22,23]. In bone tissue engineering, calcium phosphates have been used as scaffold materials. In this study, we have fabricated three-dimensional apatite-fiber scaffold with well-controlled pore sizes and porosity. Previous studies show that pore size at the range of 200–350 m is found to be optimum for bone tissue in-growth. Additionally, scaffolds involving both micro and macro porosities can perform better than only macro porous scaffold [22]. Our scaffold has been constructed with different pore size, pore-size distribution, pore type (closed or open pore) and interconnectivity. Additionally, fiber-shaped HAp, with a preferred orientation to the *c*-axis, can adsorb the protein including CTGF [24].

One of the major strategies is the delivery of signaling molecules (e.g., drugs, proteins and DNA) that promote cell migration, proliferation and differentiation. In the present study, to construct engineered bone with a network of blood vessels, we designed the three-dimensional scaffold with the ability of osteogenesis and angiogenesis by loading CTGF [14]. The CTGF is a member of the CCN family of proteins, and it interacts with cell surface molecules, growth factors, and ECM to control various kinds of cellular functions including osteogenesis and angiogenesis [25]. In fact, the CTGF stimulates osteoblast proliferation, matrix production, and differentiation in cultures of osteoblasts [26].

In the case of the CTGF-HAp or CTGF-AFS, CTGF mainly adsorbs HAp or AF via electrostatic interaction. Due to the adsorption of CTGF on ceramics or scaffold, cellular adhesion and migration are promoted compared to controls (without CTGF). A previous study demonstrated that α_v_β_1_ integrin is the primary osteoblast receptor for CTGF and that the binding site for osteoblast adhesion is involved in CTGF [17]. CTGF is believed to serve as an adhesive substrate for cells and a molecular bridge between another ECM. Another study showed that CCN2/CTGF effectively promoted the attachment of hBMSCs via its integrin α_v_β_3_ receptors [27]. Integrin binding to the ECM leads to the recruitment and phosphorylation of focal adhesion kinase (FAK) and activation of several kinases. Specifically, the ECM/integrin interaction leads to the activation of mitogen-activated protein kinase (MAPK) resulting in increased expression of osteoblast-specific genes [28]. These data let us interpret that CTGF would bind to osteoblast surface integrin and stimulate cell adhesion and migration via the MAPK signaling pathway. On the other hand, Zongjian and his colleagues reported that 13hosphor-p38 MAPK was present in pseudopodia, localizing activation of this signaling pathway to this protrusive membrane structure [29]. Taken together, the CTGF-HAp ceramics and CTGF-AFS could provide the effective matrices for osteoblasts, and formations of focal adhesions containing integrins make it possible to activate the FAK/ERK signaling pathway [17].

As for osteogenic differentiation, bone morphogenetic proteins (BMPs) and Wnt signaling play an important role to control both osteoblast differentiation and bone formation. A previous study demonstrated that CTGF was enhanced by Wnt-3A and BMP-9 at the initial stage of osteoblast differentiation [30]. Furthermore, CTGF could be modulated by members of the TGF-β superfamily, including BMPs. According to the effects of CTGF on osteoblast differentiation, CTGF is one of the good candidates for application to bone formation. Kikuchi et al. showed that CTGF incorporated hydrogel enhanced bone regeneration compared with control gel [31]. These data revealed that CTGF is an effective biomolecule for therapeutic approaches.

Furthermore, the expression of CTGF in endothelial cells is high during development, indicating a role in angiogenesis [12]. CTGF binds to integrin α_v_β_3_, which is expressed in endothelial cells and pericytes to promote endothelial cells migration and proliferation. In mice, Fisp12/mouse connective tissue growth factor accelerates the attachment of endothelial cells via the integrin receptor α_v_β_3_. Furthermore, Fisp12 stimulates the migration of endothelial cells in culture, also via an integrin- α_v_β_3_-dependent mechanism [32]. These data suggest that CTGF plays an important role in cell adhesion, migration, and survival of endothelial cells during blood vessel growth. In our in vivo study, cells could adhere to the CTGF, which were adsorbed AFS through the integrin receptor α_v_β_3_ and where cells accelerated migration and proliferation in the scaffold. Osteoblasts and endothelial cells can connect to each other, and cell-cell interactions may promote osteogenesis, as well as enhance angiogenesis. However, additional studies are warranted to elucidate the interaction between osteogenic differentiation and angiogenesis.

In summary, the CTGF-AFS could enable to regenerate bone tissue by stimulation of ECM/integrin interaction in optimally performing bone scaffold.

## 5. Conclusions

There are several important steps, such as cell attachment, migration, penetration, proliferation, and differentiation, involved in the construction of biomimetic-engineered bone. In addition, induction of vascularization into new tissue is also a key step for the clinical applicability of tissue engineering. In this study, we focused on CTGF which could fulfill the required conditions of bone regeneration, including a variety of cellular functions. Additionally, the CTGF has the vascularization ability, as well as bone formation ability which needs to construct a 3D organization. By combination with CTGF and three-dimensional porous scaffolds (AFS), we have fabricated biomimetic microenvironment and evaluated in vitro and in vivo responses. In vitro studies show that CTGF could promote cell attachment and cell migration via integrin-mediated signal transduction pathways, which would be important for subsequent cell behavior. In the case of 2D ceramics, immobilized CTGF could stimulate osteoblasts and enhance cell attachment and cell differentiation. On the other hands, osteoblasts and endothelial cells in the CTGF-AFS also were influenced by released and immobilized CTGF. On the other hand, in vivo studies show that angiogenesis and osteogenesis were significantly enhanced in the CTGF loaded-AFS group, compared to the control group of AFS without CTGF. Consequently, CTGF enhanced bone formation due to stimulation of osteogenic and angiogenic activity. These approaches show great promise for the enhancement of the functionality and clinical applicability of bone tissue engineering constructs.

## Figures and Tables

**Table 1:** The material properties of apatite-fiber scaffolds (AFSs).

-	AFS300	AFS500
Porosity/%	88.3 ± 0.25	92.8 ± 0.36
Compressive strength/MPa	0.46 ± 0.074	0.096 ± 0.018
Water adsorption rate/%	157.23 ± 9.52	356.59 ± 18.52

## Supplementary Materials

The following are available online at https://www.mdpi.com/1996-1944/12/13/2068/s1, Figure S1: Release kinetics of CTGF from CTGF-HAp ceramics, Figure S2: Release kinetics of CTGF from CTGF-AFS, Figure S3: Enhancement of cell penetration on CTGF-loaded AFS, Figure S4: Enhancement of cell penetration on CTGF-loaded AFS(500), Figure S5: Immunohistochemical analysis of angiogenesis.

## Abbreviations

CTGF	Connective tissue growth factor
HAp	Hydroxyapatite
AFS	Apatite-fiber scaffold
