# Supplementary material for: Acceleration of Osteogenesis via Stimulation of Angiogenesis by Combination with Scaffold and Connective Tissue Growth Factor
Source: Materials (Basel). 2019 Jun 27;12(13):2068. doi: 10.3390/ma12132068 (PMC6651084; doi:10.3390/ma12132068)
Supplement: Supplementary file 1 [file materials-12-02068-s001.pdf]

materials

Supplemental Data

CTGF release kinetics

CTGF release from CTGF-loaded ceramics and scaffolds was evaluated by an in vitro assay. For this purpose, CTGF-loaded samples were placed into 1 ml PBS. The supernatants were removed after one day and new buffer was added, this procedure was repeated after appropriate period. CTGF levels in the supernatant were determined with an ELISA kit (Human CTGF Standard ABTS ELISA Development Kit, PEPROTECH) according to the manufacturer's protocol.

Fig. S1 Release kinetics of CTGF from CTGF-HAp ceramics

To examine the release of CTGF from ceramics, CTGF-ceramics were immersed into 1 ml of PBS and allowed to set at 24 well plate. A rapid initial release (early burst within 24 h) is followed by a slower release (Fig. S1). About 0.05% of CTGF was released from both CTGF-HAp(50) and (100) ceramics. CTGF mainly adsorbs HAp via electrostatic interaction

Fig. S2 Release kinetics of CTGF from CTGF-AFS

To examine the release of CTGF from AFS, CTGF-AFSs were immersed in 1 ml of PBS. CTGF-released from the CTGF-loaded AFS was observed at desire time points (Figure S3). About 60 $ng \cdot cm^{-3}$ of CTGF and 50 $ng \cdot cm^{-3}$ of CTGF were released from CTGF-AFS300 and CTGF-AFS500, respectively. The CTGF-loaded AFSs continued to release CTGF over 14 days. These results suggests that the loaded CTGF could easily release in PBS depending on the porosity of AFS.

Cell penetration

MG-63 cells and HUVECs were seeded together in CTGF(0)-AFS300 or CTGF(100)-AFS300 in endothelial cell medium at an initial cell number of 2×10^5 cells and 8×10^5 cells, respectively. Cells were co-cultured for 14 days and stained with CD31 and DAPI. The distances from the surface of AFS were measured by ImageJ. Eight points from different three samples were chosen at random.

Fig. S3 Enhancement of cell penetration on CTGF-loaded AFS

The distances from the surface of CTGF(0)-AFS300 or CTGF(100)-AFS300 were measured by ImageJ. CTGF-loaded AFS could promote the cell penetration. However, no significant difference was seen between two scaffolds.

Fig. S4 Enhancement of cell penetration on CTGF-loaded AFS(500)

Cells in AFS(500) were co-cultured for 14 days and were discriminated between osteoblasts and endothelial cells by immunostaining with CD31 as a marker of endothelial cells. The number of HUVECs in CTGF(100)-AFS500 was as almost same as that in CTGF(0)-AFS500. However, micro capillary was formed on CTGF(100)-AFS500. These data suggest that cells could penetrate into AFS(500) and form three-dimensional cell-cell network. In addition, CTGF enhanced microtube formation.

In vivo study

We evaluated the ability of angiogenesis by CTGF-loaded AFSs *in vivo*. Samples were implanted into subcutaneous tissue of rats for 2 weeks and employed for histological evaluation. As a result, H&E staining showed that cells penetrated into macro pores, and well-proliferated in the CTGF(1000)-AFS500 (Fig. S5, H&E).

To assess the ability of angiogenesis by CTGF-loaded AFS, angiogenesis specific markers (CD31 and VEGF) were examined by immunostaining. Immunohistochemistry clearly showed that the

expression of CD31 in CTGF(1000)-AFS500 increased dramatically compared to AFS500. These data was agreement with AFS300. However, due to high porosity of AFS500, cells could well proliferate in AFS500 rather than AFS300.

Fig. S5 Immunohistochemical analysis of angiogenesis.

AFS and CTGF-loaded AFS showed a comparable amount of penetrated cells inside the scaffold. CTGF(1000)-AFS500 displayed a higher density of microvessels, which was further increased by CTGF loading. Formation of new microvessels was confirmed by immunohistochemical detection of CD31 and VEGF as a marker of endothelial cells (Brown DAB staining). Arrows indicate CD31 and VEGF-positive cells. Scale bars indicate 500 μm, respectively.

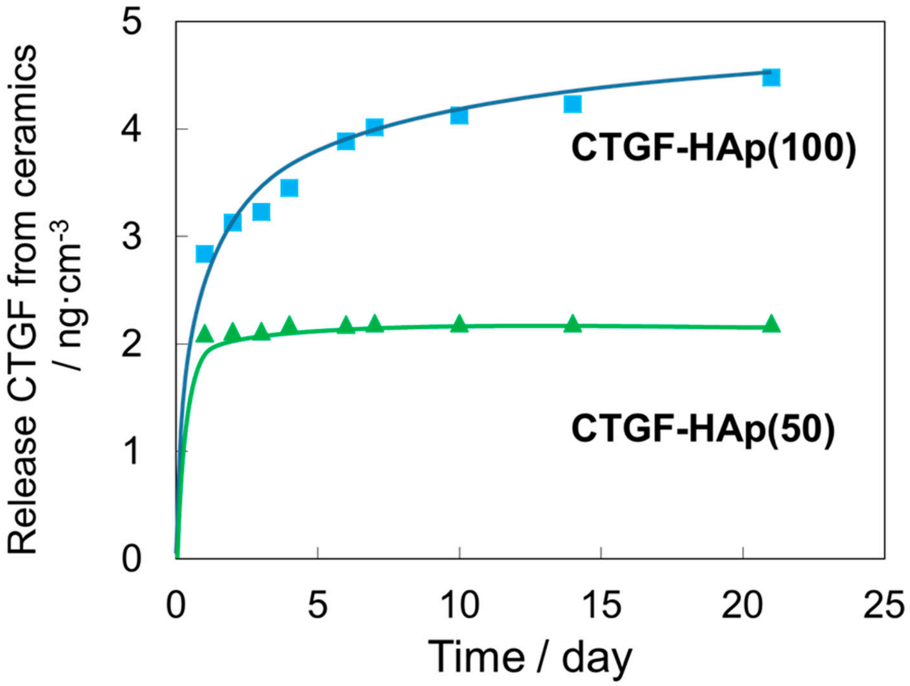

Fig. S1 Honda et al

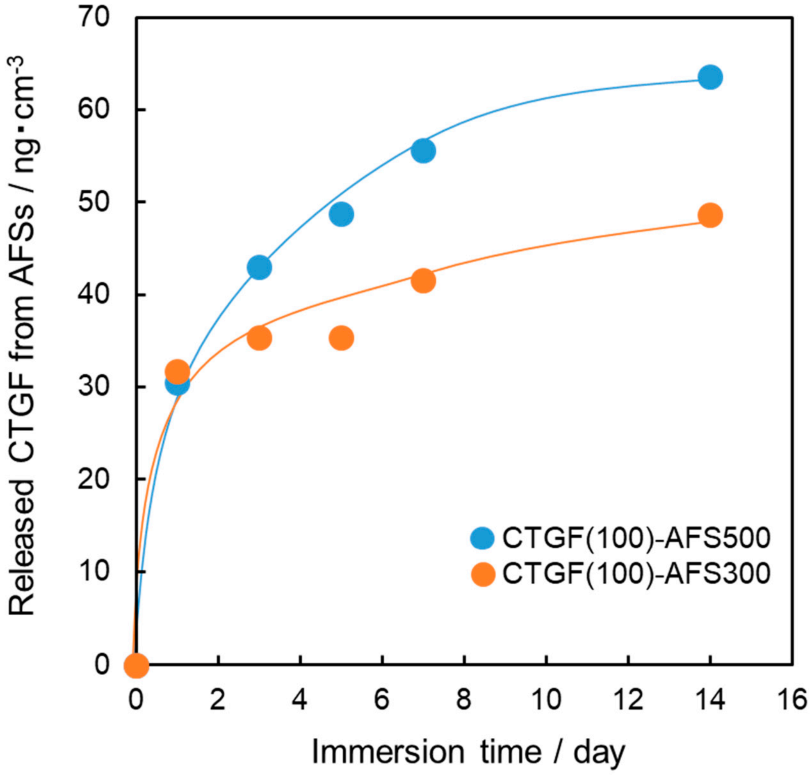

Fig. S2 Honda et al

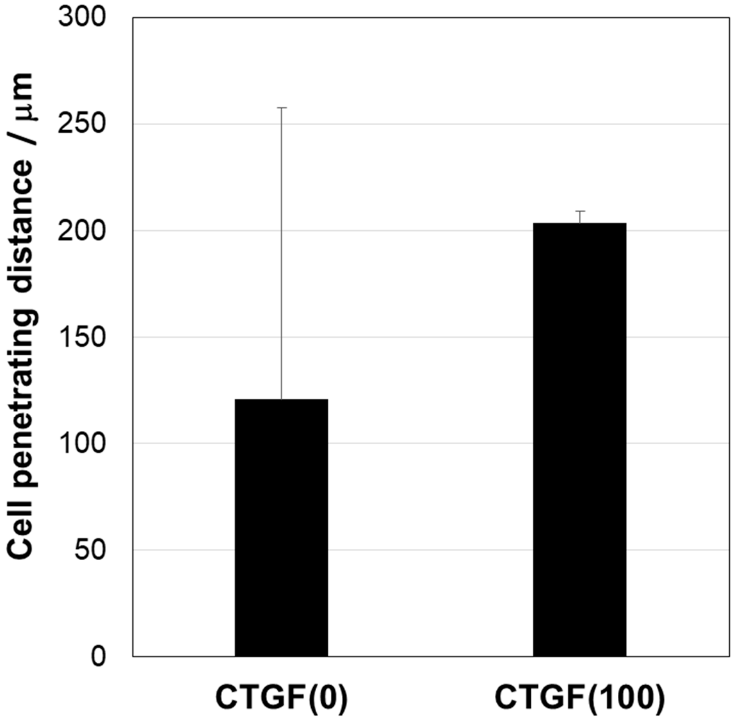

Fig. S3 Honda et al

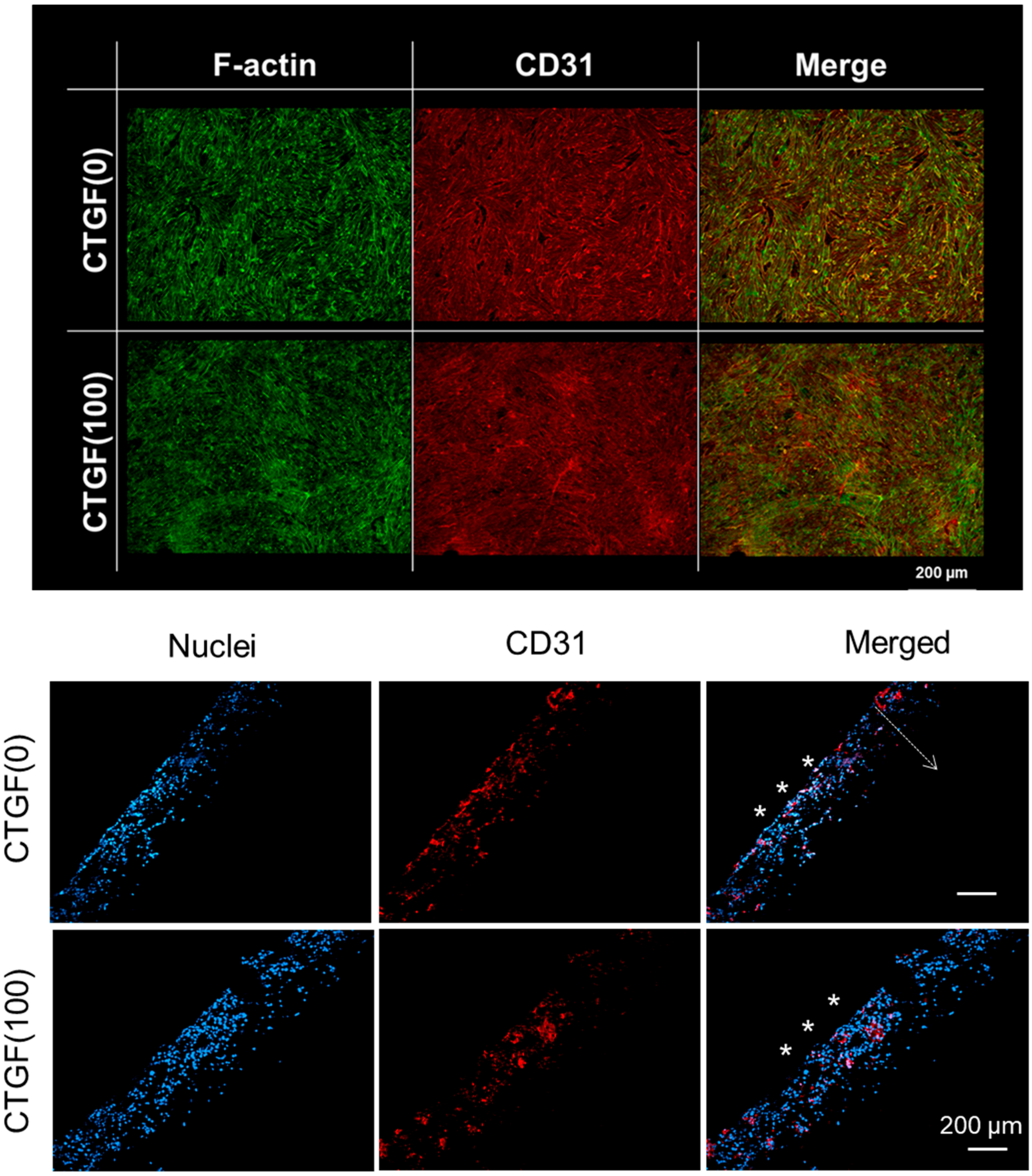

Fig. S4 Honda et al

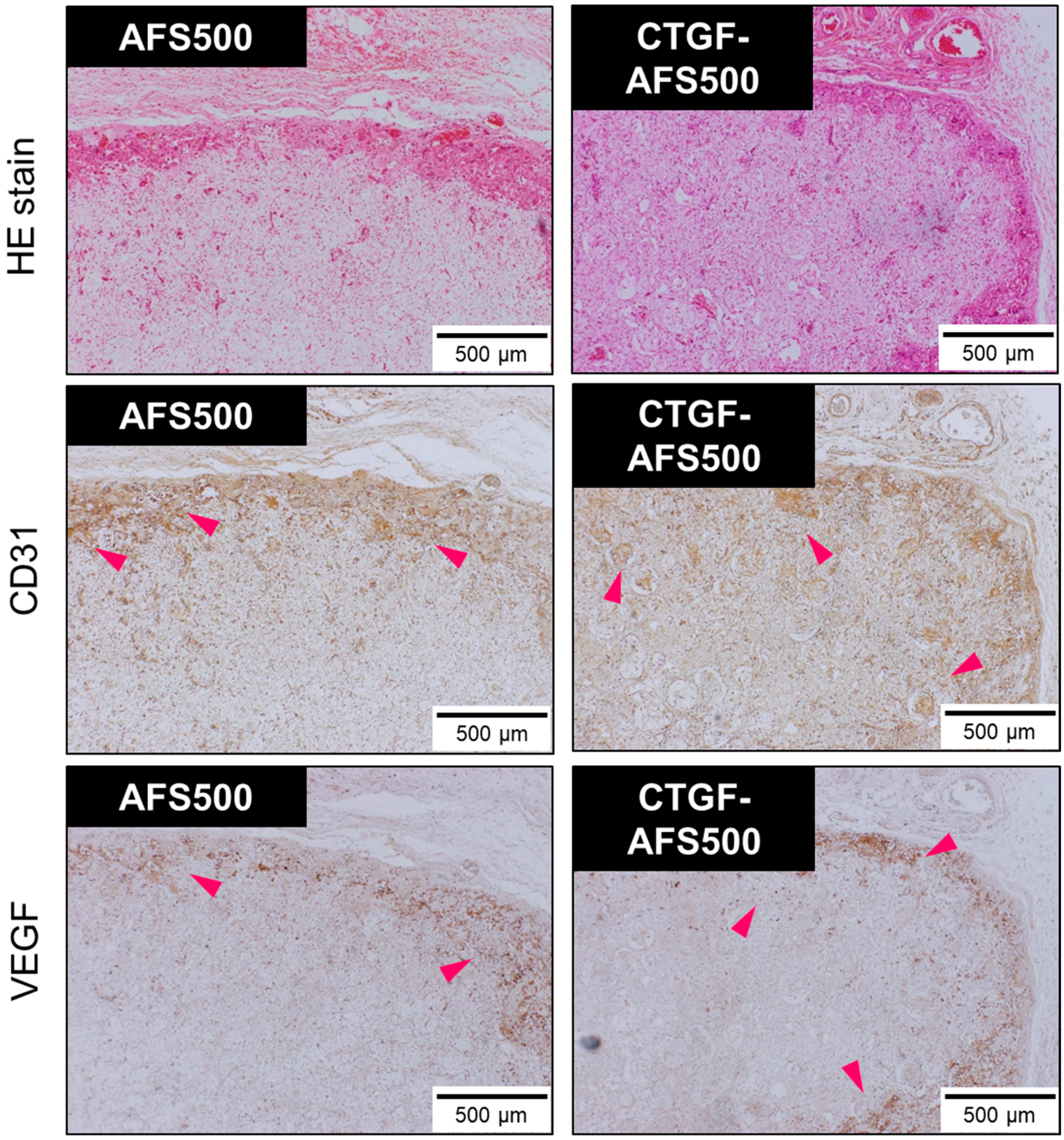

Fig. S5 Honda et al
